# Association of Kinesiophobia with Catastrophism and Sensitization-Associated Symptoms in COVID-19 Survivors with Post-COVID Pain

**DOI:** 10.3390/diagnostics13050847

**Published:** 2023-02-23

**Authors:** Manuel Herrero-Montes, César Fernández-de-las-Peñas, Diego Ferrer-Pargada, Sheila Izquierdo-Cuervo, Beatriz Abascal-Bolado, Juan Antonio Valera-Calero, Paula Paras-Bravo

**Affiliations:** 1 Departamento de Enfermería, Universidad de Cantabria, 39005 Santander, Spain; 2 Instituto de Investigación Sanitaria Valdecilla (IDIVAL), Grupo de Investigación en Enfermería, 39005 Santander, Spain; 3 Department of Physical Therapy, Occupational Therapy, Physical Medicine and Rehabilitation, Universidad Rey Juan Carlos, 28922 Madrid, Spain; 4 Servicio de Neumología, Hospital Universitario Marqués de Valdecilla, 39008 Cantabria, Spain; 5 Department of Radiology, Rehabilitation and Physiotherapy, Universidad Complutense de Madrid, 28040 Madrid, Spain; 6 Grupo InPhysio, Instituto de Investigación Sanitaria del Hospital Clínico San Carlos (IdISSC), 28040 Madrid, Spain

**Keywords:** COVID-19, pain, post-COVID, kinesiophobia, sensitization, catastrophism

## Abstract

Pain symptoms after the acute phase of severe acute respiratory syndrome coronavirus-2 (SARS-CoV-2) are present in almost 50% of COVID-19 survivors. The presence of kinesiophobia is a risk factor which may promote and perpetuate pain. This study aimed to investigate variables associated with the presence of kinesiophobia in a sample of previously hospitalized COVID-19 survivors exhibiting post-COVID pain. An observational study was conducted in three urban hospitals in Spain, including one hundred and forty-six COVID-19 survivors with post-COVID pain. Demographic (age, weight, height), clinical (intensity and duration of pain), psychological (anxiety level, depressive level, sleep quality), cognitive (catastrophizing), sensitization-associated symptoms, and health-related quality of life variables were collected in 146 survivors with post-COVID pain, as well as whether they exhibited kinesiophobia. Stepwise multiple linear regression models were conducted to identify variables significantly associated with kinesiophobia. Patients were assessed a mean of 18.8 (SD 1.8) months after hospital discharge. Kinesiophobia levels were positively associated with anxiety levels (r: 0.356, *p* < 0.001), depression levels (r: 0.306, *p* < 0.001), sleep quality (r: 0.288, *p* < 0.001), catastrophism (r: 0.578, *p* < 0.001), and sensitization-associated symptoms (r: 0.450, *p* < 0.001). The stepwise regression analysis revealed that 38.1% of kinesiophobia variance was explained by catastrophism (r^2^ adj: 0.329, *B =* 0.416, t = 8.377, *p* < 0.001) and sensitization-associated symptoms (r^2^ adj: 0.381, *B =* 0.130, t = 3.585, *p* < 0.001). Kinesiophobia levels were associated with catastrophism and sensitization-associated symptoms in previously hospitalized COVID-19 survivors with post-COVID pain. Identification of patients at a higher risk of developing a higher level of kinesiophobia, associated with post-COVID pain symptoms, could lead to better therapeutic strategies.

## 1. Introduction

Up to 60% of subjects who have survived to the severe acute respiratory syndrome coronavirus-2 (SARS-CoV-2), the agent causing coronavirus disease, 2019 (COVID-19), can experience a plethora of symptoms after the acute phase of the infection, i.e., post-COVID or long COVID [1]. Different meta-analyses have identified the presence of more than 50 post-COVID symptoms, e.g., fatigue, dyspnea, memory loss, brain fog, ageusia, anosmia, which can be present months [2,3], and up to one year [4,5,6], after infection. Pain is a post-COVID symptom experienced during the first year after the infection, with a pooled prevalence of 20% when investigated as a general symptom [7], or up to 60% when specifically assessed [8,9,10,11]. Different studies have reported a heterogeneous location of post-COVID pain, with a prevalence of 20–25% for widespread distribution [12,13,14]. The presence of widespread pain is associated with altered nociceptive processing, which has been recently identified in individuals with post-COVID pain [15].

Chronic pain is a complex condition where biological, cognitive, behavioral, and social aspects contribute to its clinical presentation, as maladaptive psychological factors influence the pain experience. In fact, fear of movement, also known as kinesiophobia, is considered a relevant factor influencing the chronification, persistence, and deterioration of pain [16]. There is evidence that higher levels of kinesiophobia are associated with greater pain intensity and related disability (strong evidence), and with lower quality of life (moderate evidence) [17]. In addition, fear-avoidance behaviors are also associated with other cognitive factors, e.g., pain catastrophism and hypervigilance [18,19], which could result in a significant decrease in activity. Since physical activity is a protective behavior against chronic pain [20,21], and it is being advocated as an important strategy against post-COVID symptoms [22], identifying kinesiophobic behaviors in patients with chronic pain is highly recommended for screening subjects who may show reduced adherence to active treatments, due to an irrational and excessive fear of performing physical activity [23].

Despite a high prevalence of kinesiophobia in musculoskeletal pain conditions [24], and the association between physical inactivity and a higher risk for severe COVID-19 [25], current evidence assessing whether COVID-19 survivors with post-COVID also exhibit these maladaptive cognitive behaviors is limited. Since the COVID-19 outbreak, associated factors have increased stress, anxiety, fear, and physical inactivity [26]. Analyzing whether kinesiophobic behavior is present in COVID-19 survivors with post-COVID pain, as well as its association with sensitization-associated symptoms, catastrophism, and other features, will be of high interest. This study aimed to investigate the association between kinesiophobia levels and other pain-related mechanisms, e.g., sensitization-associated symptoms, catastrophism, and mood disturbances, in individuals with long-term post-COVID pain. Since being female has been found to be a risk factor associated with post-COVID pain [12], the study was conducted from a gender perspective. We hypothesized that those individuals with higher levels of anxiety and depression, and higher sensitization-associated symptoms, would exhibit higher levels of kinesiophobia.

## 2. Methods

### 2.1. Study Design

An observational cross-sectional cohort study, following the Strengthening the Reporting of Observational studies in Epidemiology (STROBE) guidelines [27], was conducted. This study was approved by the Local Institutional Ethics Committees (INDIVAL Cantabria 2020.416; HUIL/092-20, HUFA 20/126URJC0907202015920; HSO25112020). Patients were informed of the study and all provided their written informed consent prior to their inclusion.

### 2.2. Participants

Individuals who had recovered from acute SARS-CoV-2 infection at three urban hospitals in Spain (Hospital Universitario Infanta Leonor, Hospital Universitario Fundación Alcorcón, and Hospital Severo Ochoa), during the first wave of the COVID-19 pandemic, were screened for eligibility. The inclusion criteria were: (i) diagnosis of acute SARS-CoV-2 infection by real-time reverse transcription-polymerase chain reaction (RT-PCR) assay of nasopharyngeal, or oral swab sample, and the presence of consistent clinical and radiological findings at hospitalization; (ii) reporting “de novo” pain symptoms starting after the infection and lasting for at least three consecutive months; and (iii) absence of any potential underlying medical condition which could best explain the pain, e.g., arthritis. Although post-COVID pain exhibits mixed features of musculoskeletal and neuropathic pain, we defined post-COVID pain compatible with the diagnosis of chronic primary musculoskeletal pain as defined by the International Association for the Study of Pain (IASP) [28]. Exclusion criteria included: (i) previous history of pain symptoms before the infection; and (ii) any other pre-existing medical comorbidity explaining pain symptoms.

A structured questionnaire, including clinical data of their pain and several patient-reported outcome measures (PROMs), was used for data collection. Age, weight, height, and intensity (numerical pain rating scale, NPRS, 0–10) and duration of pain symptoms were collected. The PROMs evaluated sensitization-associated symptoms, neuropathic pain features, anxiety levels, depression levels, sleep quality, catastrophism, and health-related quality of life. In addition, kinesiophobia was used as the primary outcome of the study.

### 2.3. Kinesiophobia

The main dependent outcome of this study was kinesiophobia (fear of movement), defined as an excessive, irrational, and debilitating fear to perform a physical movement, due to a feeling of vulnerability to a painful injury or reinjury [17]. We used the 11-item Tampa Scale for Kinesiophobia (TSK-11) for evaluating the fear of movement [29]. This specific PROM consists of 11 questions, where the patient chooses how much they agree or disagree with each item, 1 being “complete disagreement”, and 4 “complete agreement” (total score from 11 to 44) [30].

Although no clear cutoff score is considered for the TSK-11, we adapted the score proposed by Nicholas et al. for the TSK-17 in different chronic pain conditions [30]. Accordingly, kinesiophobia was considered as minimal (TSK-11 score ≤ 22), low (TSK-11 from 23 to 28), moderate (TSK-11 from 29 to 35), or high (TSK-11 ≥ 36).

### 2.4. Sensitization-Associated Symptoms

The Central Sensitization Inventory (CSI) was used to evaluate the presence of sensitization-associated symptoms. It includes 25 health-related symptoms assumed to represent aspects of sensitization, each based on 5-point Likert scale rating [31]. The score ranges from 0 to 100, where > 40 points suggest the presence of sensitization-associated symptoms [32]. The CSI has shown good psychometric properties for assessing sensitization-associated symptoms in patients with persistent pain [33,34]. Previous studies using the CSI in individuals with post-COVID symptoms have reported conflicting results, since Goudman et al. found that 70% of individuals with long COVID exhibited a CSI score ≥ 40/100 points [35], whereas Fernández-de-las-Peñas et al. found that just 34% of patients with post-COVID pain exhibited a CSI score ≥ 40/100 points [36].

### 2.5. Psychological/Cognitive Variables

The Hospital Anxiety and Depression Scale (HADS) was used to evaluate anxiety (HADS-A, 7-items) and depression (HADS-D, 7-items) levels [37]. The total score of each subscale ranges from 0 to 21 points, where ≥12 points on the HADS-A is indicative of anxiety symptoms, and ≥10 points on the HADS-D indicates depressive symptoms [38]. It has been observed that both scales of this questionnaire (HADS-A and HADS-D) exhibit good psychometric properties to be used for assessing psychological and emotional stress in COVID-19 survivors with long COVID [39].

Sleep quality was assessed with the Pittsburgh Sleep Quality Index (PSQI) [40]. This PROM consists of 19 self-rated questions (rated from 0 to 3) assessing different aspects of sleep (e.g., usual bedtime, wake-up time, number of hours slept, and time needed to fall asleep) during the previous month. The total score ranges from 0 to 21 points, where ≥8.0 points are indicative of being a poor sleeper [40].

Pain catastrophizing, i.e., an exaggerated negative mental state brought to bear during an actual or anticipated painful experience, was assessed with the Pain Catastrophizing Scale (PCS). This PROM includes 13-items (rated 0: never, to 4: always) evaluating rumination, magnification, and despair aspects in relation to the pain experience. The total score ranges from 0 to 52 points [41].

### 2.6. Health-Related Quality of Life

The paper-based five-level version of EuroQol-5D-5L (EQ-5D-5L) was used to assess health-related quality of life [42]. This questionnaire assesses mobility, self-care, daily activities, pain, and depression/anxiety dimensions from 1 (no problems) to 3 (severe problems) points. Responses were converted into a single index number between 0 and 1 where 0 corresponds to a health state judged to be equivalent to death and 1 corresponds to optimal health, by applying crosswalk index values for Spanish life [43]. This questionnaire has exhibited good psychometric properties to be used as a PROM to assess health-related quality of life in hospitalized COVID-19 survivors with long-COVID [44].

### 2.7. Sample Size Determination

Austin and Steyerberg suggested that linear regression models require only two subjects per variable SPV for adequate estimation of coefficients [45]; however, this simple calculation would lead to small sample sizes. Recently, Jenkins and Quintana-Ascencio [46] proposed that an adequate sample size for regression models should include between 10 and 15 subjects per variable, and no more than five predictors within the model. Accordingly, for five potential predictor variables, a minimum of 75 participants would be required. A statistical calculation using the G*Power software v.3.1. (Heinrich-Heine-Universität Düsseldorf, Düsseldorf, Germany) was also performed, setting a *t*-test for a linear multiple regression fixed model with a single regression coefficient. Setting two tails, with a standard effect size of 0.015, an alpha error of 0.05, a statistical power of 0.95, and two predictors, a sample size of 89 participants would result in an adequate statistical power (>0.95). To identify the highest number of variables that could be associated with kinesiophobia, and for avoiding potential type II errors, we significantly increased the estimated sample size.

### 2.8. Statistical Analysis

Descriptive analyses (means and standard deviations (SD)) were used to describe the samples. The Kolmogorov–Smirnov test revealed that all quantitative data exhibited a normal distribution. Between-gender differences were initially assessed with independent Student t-tests. First, correlations between all variables and the dependent variable (TSK-11) were initially assessed by using Pearson correlation coefficients (r). The correlation analysis was used to identify multicollinearity and shared variance between the variables (r > 0.8). Second, statistically significant variables associated with TSK-11 were included into a stepwise multiple linear regression model (i.e., a hierarchical regression analysis), to identify those independent variables contributing significantly to the variance of the TSK-11, except the variables showing multicollinearity. The significance criterion of the F value for entry into the regression equation was set at *p* < 0.05. Changes in the adjusted R^2^ were reported after each step of the regression model, to determine the association of the additional variables.

## 3. Results

From 200 patients with post-COVID symptoms screened for participation, finally, 146 (73%) fulfilled all criteria and agreed to participate. They were assessed a mean of 18.8 ± 1.8 months after hospital discharge. Fifty-four patients were excluded because their main post-COVID symptom was fatigue or dyspnea, but not pain. Table 1 details the demographic, clinical, sensory-related, quality of life, and psychological features of the total sample, and by gender. The males were older (*p* = 0.013), and had greater heights (*p* < 0.001) and weights (*p* = 0.002) than the females. Further, pain intensity (*p* = 0.016), sensitization-associated symptomatology (CSI, *p* < 0.001), sleep quality (PSQI, *p* < 0.001), anxiety levels (HADS-A, *p* = 0.02), and kinesiophobia (TSK-11, *p* = 0.044) were significantly higher in females when compared with males. Sixteen (10.9%) patients exhibited high kinesiophobia levels, 31 [21.3%) had moderate levels, 36 [24.6%) low to moderate levels, and the remaining 63 (43.2%) had minimal kinesiophobia levels.

### 3.1. Bivariate Correlation Analyses

Table 2 summarizes the bivariate correlation analyses. Kinesiophobia levels (TSK-11) were positively associated with anxiety (r = 0.356, *p* < 0.001) and depression (r = 0.306, *p* < 0.001) levels, sleep quality (r = 0.288, *p* < 0.001), sensitization-associated symptoms (r = 0.450, *p* < 0.001), and catastrophism (r = 0.578, *p* < 0.001): higher levels of kinesiophobia were associated with higher anxiety/depression levels, worse quality of sleep, higher sensitization-associated symptomatology, and higher pain catastrophizing. Post-COVID symptom duration, pain intensity, and health-related quality of life did not show significant correlation with kinesiophobia levels.

In addition, other associations were also found: (i) pain intensity was associated with female sex (*p* < 0.05), higher anxiety (*p* < 0.05), depression (*p* < 0.01) and sensitization-associated symptoms (*p* < 0.05); (ii) both anxiety and depression levels were associated with higher catastrophism (*p* < 0.01), sensitization-associated symptoms (*p* < 0.01), and poorer quality of life (*p* < 0.05 for HADS-A; *p* < 0.01 for HADS-D).

**Figure 1:**
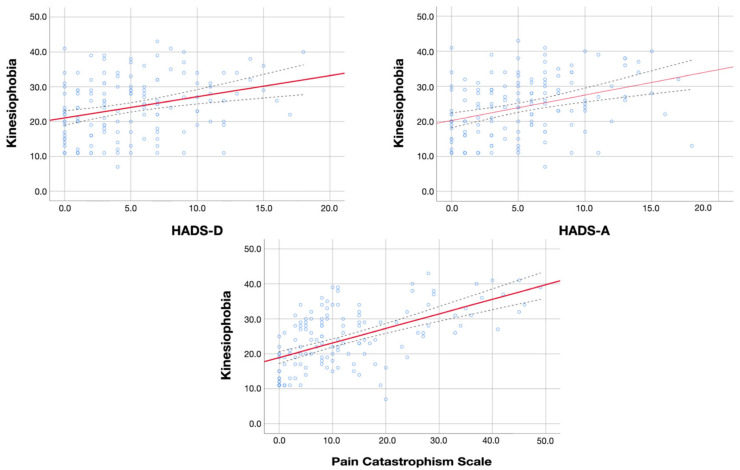
Association between kinesiophobia and psychological variables: anxiety (HADS-A), depression (HADS-D) and catastrophism. Red line represents the correlation coefficient whereas blue dot lines represent the confidence intervals of the coefficient.

**Figure 2:**
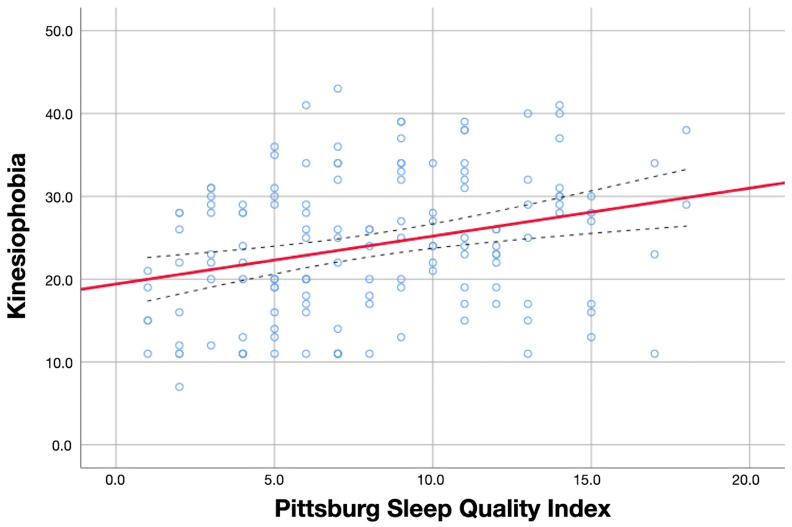
Association between kinesiophobia and sleep quality. Red line represents the correlation coefficient whereas blue dot lines represent the confidence intervals of the coefficient.

**Figure 3:**
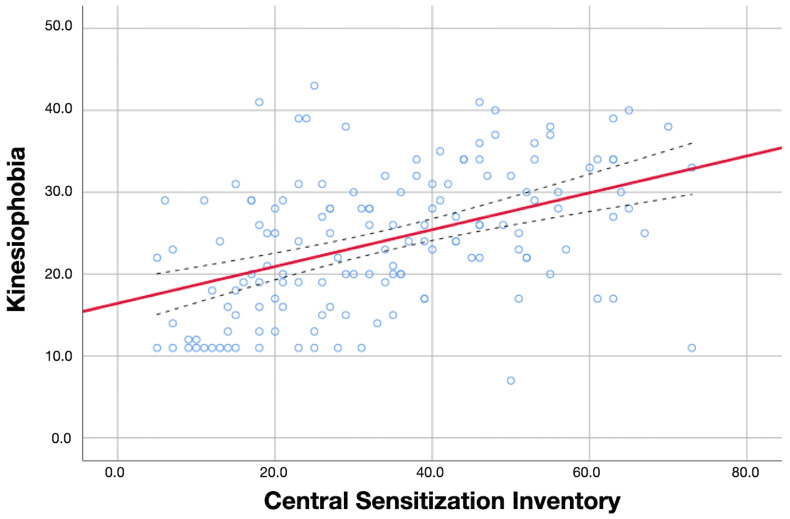
Association between kinesiophobia and sensitization-associated symptoms (CSI). Red line represents the correlation coefficient whereas blue dot lines represent the confidence intervals of the coefficient.

Additionally, the associations of kinesiophobia levels with psychological variables, sleep quality, and sensitization-associated symptoms are illustrated in Figure 1, Figure 2 and Figure 3, respectively.

### 3.2. Multiple Regression Analyses

**Figure 4:**
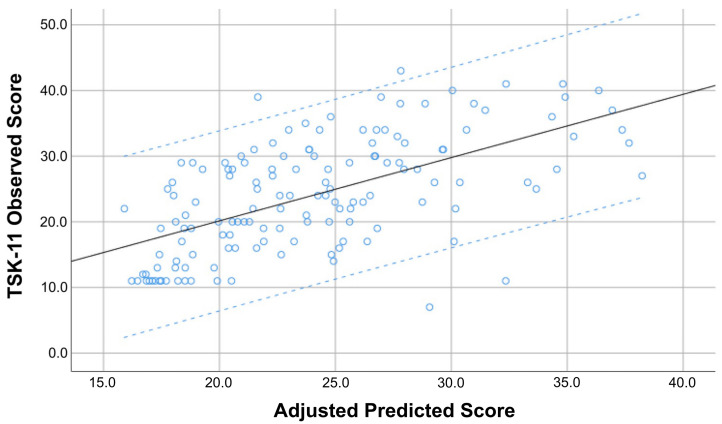
Scatter plot of the adjusted predicted score (r^2^ adjusted: 0.381) explaining kinesiophobia levels (TSK-11) score in previously hospitalized COVID-19 survivors exhibiting “de novo” post-COVID pain symptoms (*n* = 146). Note that some points can be overlapping.

The hierarchical regression analysis to determine the explained variance of the TSK-11 score is summarized in Table 3. Stepwise regression analyses revealed that the PCS score (contributing 32.9%), and CSI score (contributing an additional 5.2%) were significantly associated, and combined explained 38.1% of the variance for the TSK-11 score (r^2^ adjusted: 0.381, Figure 4).

## 4. Discussion

This study used regression analyses for investigating which factors may contribute to the variance of kinesiophobia levels in individuals exhibiting “de novo” post-COVID pain. Kinesiophobia was associated with catastrophism and sensitization-associated symptoms in previously hospitalized COVID-19 survivors with post-COVID pain. In addition, the overall prevalence of kinesiophobia in people with chronic pain ranges from 50% to 70% [24,47]. We observed that almost 57% of COVID-19 survivors with post-COVID pain reported a potential kinesiophobic behavior. This finding highlights the importance of considering cognitive, in addition to biological, factors, to explain post-COVID pain.

Pain processing and pain-related information in people with chronic pain could be related to how kinesiophobia is perceived. In fact, the association between kinesiophobia and pain catastrophism supports that the fear-avoidance model could be also applied to people with post-COVID pain. The fear-avoidance model proposes that a catastrophic misinterpretation of pain would lead to higher fear of movement and also hypervigilance, leading to potential maladaptive avoidance behavior resulting in reduced function, disuse, and increased symptoms [48]. In fact, pain catastrophizing can impact the central nervous system by amplifying pain related signals, influencing descending pain inhibition, and by behavioral pathways, leading to an inability to control pain-related thoughts [49]. These maladaptive behaviors could provoke a vicious cycle perpetuating pain. The fear-avoidance model is supported by a meta-analysis showing significant associations between cognitive behaviors, i.e., kinesiophobia, catastrophizing, and pain hypervigilance, with pain intensity and pain-related disability [50]. Interestingly, kinesiophobia was not directly associated with the intensity of pain in our sample of individuals with post-COVID pain, in agreement with previous studies in chronic pain conditions of the lower extremities, such as knee [51] or plantar heel pain [52], but contrary to observations in chronic postsurgical pain [53]. Luque-Suarez et al. [17] found moderate evidence supporting the association between kinesiophobia and pain intensity in musculoskeletal pain conditions. Today, we can not consider post-COVID pain as a musculoskeletal pain condition, which could explain the lack of association between kinesiophobia levels and the intensity of pain.

Extensive evidence supports the idea that chronic pain is associated with sensitization [54]. In fact, current data suggest that people with post-COVID pain exhibit altered pain processing (sensitization) [35,36]. We observed that kinesiophobia levels were associated with sensitization-associated symptomatology, as assessed by the CSI. The fact that psychological and cognitive factors are associated with sensitization symptoms, agrees with previous studies in individuals with chronic pain [55]. This finding agrees with those theories supporting the idea that sensitization-associated symptoms, based on the CSI, have a significant overlap with the cognitive/psychological construct [56]. This can be explained since maladaptive cognitive behaviors, e.g., kinesiophobia and pain catastrophizing, are also considered central nervous system-derived symptoms [56]. Current findings would support that both biological and cognitive mechanisms are important for patients with post-COVID pain. In fact, the presence of kinesiophobia levels and sensitization-associated symptoms would support that post-COVID pain could be considered as a nociplastic pain condition, a hypothesis which has been recently proposed [57].

The results of this study have several clinical implications. Cognitive factors, such as pain catastrophizing and kinesiophobia, will require consideration in the management of individuals with post-COVID pain. Accordingly, clinicians managing people with post-COVID pain should listen to the patient’s history for their thoughts, feelings, and behaviors that indicate either fear of movement, rumination, magnification of the threat value of pain, or a sense of helplessness. Treatments, including cognitive behavioral interventions, are recommended for the management of kinesiophobia caused by musculoskeletal pain; however, psychological interventions, such as coping strategies, are also potentially applicable [58]. Kamonseki et al. reported that manual therapy strategies could also be equally effective as other cognitive interventions for managing cognitive maladaptative behaviors such as fear avoidance, kinesiophobia, and pain catastrophizing, again, in people with musculoskeletal pain conditions [59]. Deciding when to address these cognitive behaviors in clinical practice remains unclear, and probably they should be managed at the same time as biological factors associated with long COVID. In fact, a recent meta-analysis concluded that the clinical effects of isolated interventions, such as pain neuroscience education, are smaller than expected, at least in the short-term [60]. Since cognitive factors including kinesiophobia and pain catastrophizing are associated with sensitization-associated symptoms, clinicians should consider multimodal individually tailored treatments, combining pain neuroscience education with physical therapy and stress management.

The presence of kinesiophobia in individuals with post-COVID pain could also limit the application of exercise programs due to this maladaptive behavior. Exercise is the therapeutic option most recommended for the management of individuals with long-COVID, including those with pain symptoms [61]. Accordingly, the presence of higher levels of kinesiophobia could reduce the potential benefits of exercise, due to fear. In such a scenario, exercise should be adapted to each particular patient, and combined with cognitive behavior strategies, and applied based on a graded-exposure principle [22].

Finally, these results should be considered according to their potential limitations. First, current data can be only applicable to previously hospitalized COVID-19 survivors with mild-to-moderate severity. In fact, critically ill COVID-19 survivors also develop post-COVID pain symptoms, and the role of maladaptive cognitive behaviors could be different [62]. Second, we excluded patients with pre-existing pain symptoms before the infection, since this is a risk factor for developing post-COVID pain [7]. We do not know if the presence of pain symptoms before the infection would lead to a facilitation of these maladaptive cognitive behaviors and sensitization features. Third, we collected different PROMs with potential overlapping between them. For instance, the CSI is also able to assess psychological/emotional constructs. Finally, due to the cross-sectional nature of the design, causal relationships between these maladaptive cognitive behaviors, post-COVID pain, and sensitization-associated symptoms cannot be determined.

## 5. Conclusions

This study found that almost 60% of previously hospitalized COVID-19 survivors suffering from post-COVID pain exhibit kinesiophobia. In addition, kinesiophobia levels were associated with catastrophism and sensitization-associated symptoms. Identification of patients at a higher risk of developing higher levels of kinesiophobia associated with post-COVID pain symptoms could lead to therapeutic strategies targeting these cognitive behaviors able to promote and perpetuate pain.

## Figures and Tables

**Table 1:** Baseline outcomes (mean ± SD) of the sample.

Baseline	Sample (*n* = 146)	Males (*n* = 67)	Females (*n* = 78)	Between-Gender Differences
Demographic Characteristics				
Age (years)	57.5 ± 11.8	60.0 ± 10.3	55.2 ± 12.5	4.8 (1.03; 8.65) *p* = 0.013
Height (m)	1.67 ± 0.09	1.73 ± 0.08	1.61 ± 0.06	0.11 (0.08; 0.13) *p* < 0.001
Weight (kg)	81.8 ± 17.1	86.5 ± 15.6	77.8 ± 17.4	8.7 (3.3; 14.2) *p* = 0.002
Clinical Characteristics				
Post-COVID symptoms (months)	18.8 ± 1.8	18.7 ± 2.0	18.9 ± 1.7	0.2 (−0.4; 0.8) *p* = 0.489
Pain-Related Features				
Pain intensity (0 to 10)	5.59 ± 1.72	5.23 ± 1.85	5.92 ± 1.54	0.69 (0.13; 1.25) *p* = 0.016
CSI (0 to 100)	33.91 ± 17.25	25.92 ± 14.33	41.06 ± 16.46	15.13 (10.02; 20.24) *p* < 0.001
Quality of Life				
EuroQol-5D-5L Questionnaire (0 to 100)	0.77 ± 0.20	0.79 ± 0.22	0.76 ± 0.17	0.02 (−0.03; 0.09) *p* = 0.427
Pittsburgh Sleeping Quality Index (0 to 21)	8.07 ± 4.28	6.86 ± 4.42	9.11 ± 3.91	2.24 (0.88; 3.61) *p* = 0.001
Psychological Characteristics				
HADS-A (0 to 21)	5.28 ± 4.21	4.44 ± 4.04	6.07 ± 4.22	1.62 (−0.26; 2.99) *p* = 0.020
HADS-D (0 to 21)	5.07 ± 4.29	4.38 ± 4.28	5.60 ± 4.27	1.21 (−0.19; 2.62) *p* = 0.091
Pain Catastrophizing Scale (0 to 52)	12.14 ± 11.95	10.27 ± 11.30	13.80 ± 12.40	3.52 (−0.43; 7.48) *p* = 0.080
Tampa Scale for Kinesiophobia (0 to 44)	24.11 ± 8.56	22.59 ± 8.74	25.47 ± 8.25	2.88 (0.07; 5.68) *p* = 0.044

Abbreviations: HADS, Hospital Anxiety and Depression Scale.

**Table 2:** Pearson product-moment correlation matrix between sociodemographic, psychological, neuro-physiological, and clinical characteristics.

	1	2	3	4	5	6	7	8	9	10	11	12
1. Age												
2. Gender	−0.206 *											
3. Height	0.003	−0.595 **										
4. Weight	−0.090	−0.256 **	0.509 **									
5. Post-COVID symptoms	−0.122	0.058	0.010	0.127								
6. Pain intensity	−0.047	0.200 *	−0.191 *	−0.109	0.016							
7. HADS-A	0.028	0.194 *	−0.158	−0.090	−0.271 **	0.175 *						
8. HADS-D	0.078	0.141	−0.104	−0.091	−0.136	0.225 **	0.750 **					
9. PSQI	0.121	0.262 **	−0.213 **	−0.102	−0.189 *	0.137	0.316 **	0.354 **				
10. CSI	−0.076	0.440 **	−0.285 **	−0.121	−0.158	0.190 *	0.551 **	0.446 **	0.390 **			
11. PCS	0.132	0.147	−0.128	−0.083	−0.343 **	0.045	0.492 **	0.483 **	0.282 **	0.402 **		
12. TSK-11	0.000	0.168 *	−0.065	0.034	−0.092	0.150	0.356 **	0.306 **	0.288 **	0.450 **	0.578 **	
13. EuroQol-5D-5L	−0.039	−0.066	0.004	0.051	0.081	−0.006	−0.143	−0.174 *	−0.301 **	−0.199 *	−0.210 *	−0.132

Abbreviations: CSI, Central Sensitization Inventory; HADS, Hospital Anxiety and Depression Scale. * *p* < 0.05; ** *p* < 0.01.

**Table 3:** Summary of the stepwise regression analyses to determine predictors of TSK-11.

	Predictor Outcome	Β	SE B	95% CI	B	t	*p* Value
TSK-11	Step 1						
	Catastrophism	0.416	0.050	0.318; 00.515	0.578	80.377	<0.001
	Step 2						
	Catastrophism	0.343	0.052	0.240; 00.445	0.475	60.593	<0.001
	Central Sensitization Inventory	0.130	0.036	0.058; 00.201	0.259	30.585	<0.001

R^2^ adj. = 0.329 for step 1; R^2^ adj. = 0.381 for step 2.

## Data Availability

All data derived from the study are reported in this manuscript.
